# Potentially Same Novel *Ehrlichia* Species in Horses in Nicaragua and Brazil

**DOI:** 10.3201/eid2405.172076

**Published:** 2018-05

**Authors:** Thállitha S.W.J. Vieira, Barbara A. Qurollo, Anna C.B. Mongruel, Rafael A. Baggio, Odilon Vidotto, Edward B. Breitschwerdt, Rafael F.C. Vieira

**Affiliations:** Universidade Federal do Paraná, Curitiba, Brazil (T.S.W.J. Vieira, A.C.B. Mongruel, R.A. Baggio, R.F.C. Vieira);; North Carolina State University, Raleigh, North Carolina, USA (B.A. Qurollo, E.B. Breitschwerdt);; Universidade Estadual de Londrina, Londrina, Brazil (O. Vidotto)

**Keywords:** tickborne diseases, equine, ehrlichiosis, Nicaragua, Brazil, horses, Ehrlichia, 16S rDNA, sodb, groEL, bacteria, rickettsia, vector-borne infections

**To the Editor:** In our previously published report, we found that blood samples from 4 naturally infected horses in Nicaragua were PCR positive for the 16S rDNA, *sodB*, and *groEL* genes of an *Ehrlichia* species (*1*). Similarly, Vieira and colleagues reported a potentially novel *Ehrlichia* sp. infecting horses in South America, with a high seroprevalence in carthorses; 1 horse blood sample was PCR positive for *Ehrlichia* 16S rDNA and *dsb* genes (*2*). Because these 2 studies sequenced different 16S rDNA regions, the *Ehrlichia* sp. found in Nicaragua could not be established as the same one infecting horses in Brazil.

We retrieved an *Ehrlichia* PCR-positive horse blood sample (*2*) from Brazil and performed partial PCR and sequencing of the 16S rDNA, *sodb*, and *groEL* genes (*1*). Phylogenetic analysis of the sequences (*3*–*5*) demonstrated a close relationship between the *Ehrlichia* spp. found in Brazil and Nicaragua, with posterior probability values of 100% for all 3 gene fragments (Technical Appendix Figure 1). The 16S rDNA were 100% identical (181 bp/181 bp; GenBank accession no. KJ434178), *sodb* 99% identical (561 bp/567 bp; GenBank accession nos. MG385129, KJ434180), and *groEL* 99% identical (579 bp/584 bp; GenBank accession nos. MG385128, KJ434179). When we compared translated amino acid sequences of the *Ehrlichia* spp. from Brazil and Nicaragua, we observed high percentage identities with the *groEL* (100%) and *sodB* (97.8%) alignments (Technical Appendix Figure 2). Furthermore, when compared with *E. ruminantium*, the most closely related *Ehrlichia* sp. on the basis of phylogenetic analyses, percentage identities from the *groEL* (94.8%) and *sodB* (78.8%) alignments were lower for both *Ehrlichia* spp.

These findings suggest that the novel *Ehrlichia* spp. found infecting horses in Nicaragua and Brazil are potentially the same species. Future studies are needed to determine cell culture practices, characterize potential clinical signs of infection, and establish the main vector of this novel equine *Ehrlichia* species.

Technical Appendix Phylogenetic analysis of 16S rDNA, *groEL*, and *sodb* gene fragments and partial amino acid alignment of sodB and groEL of *Ehrlichia* isolates found in horses in Nicaragua and Brazil.
