# Supplementary material for: Potentially Same Novel Ehrlichia Species in Horses in Nicaragua and Brazil
Source: Emerg Infect Dis. 2018 May;24(5):953. doi: 10.3201/eid2405.172076 (PMC5938758; doi:10.3201/eid2405.172076)
Supplement: Technical Appendix — Phylogenetic analysis of 16S rDNA, groEL, and sodb gene fragments and partial amino acid alignment of sodB and groEL of Ehrlichia isolates found in horses in Nicaragua and Brazil. [file 17-2076-Techapp-s1.pdf]

Article DOI: https://doi.org/10.3201/eid2405.172076

# Potentially Same Novel *Ehrlichia* Species in Horses in Nicaragua and Brazil

## Technical Appendix

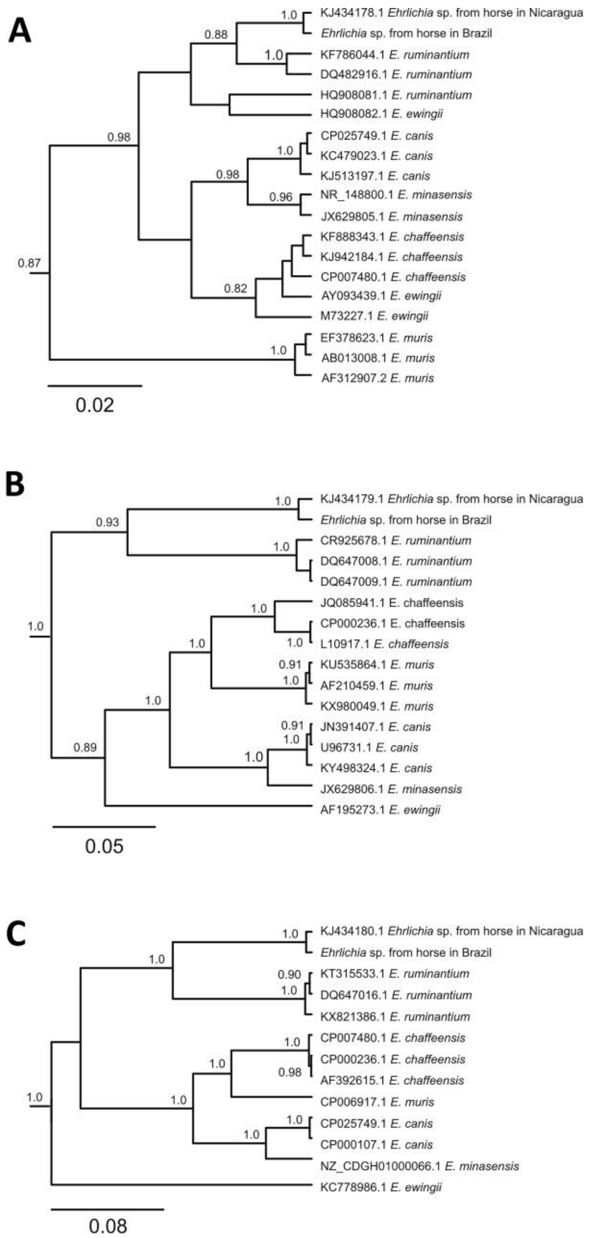


**Technical Appendix Figure 1.** Phylogenetic analysis of gene fragments of *Ehrlichia* isolates found in horses in Nicaragua and Brazil compared with reference strains. Trees were constructed by using 181 bp of 16S rDNA (A), 561 bp of *groEL* (B), and 579 bp of *sodb* (C) genes.

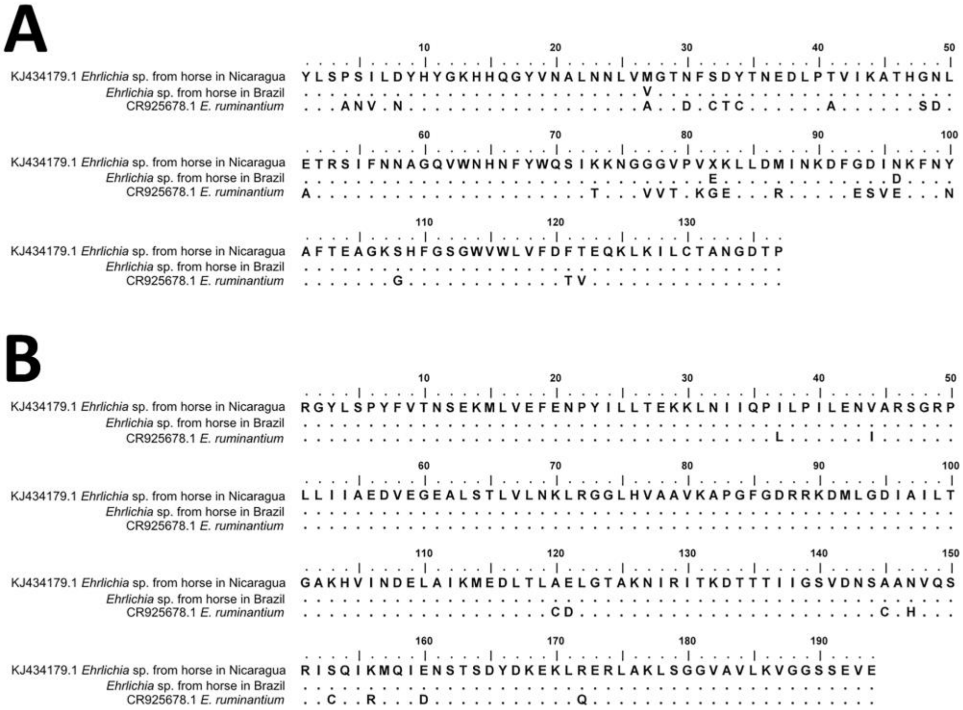

**Technical Appendix Figure 2.** Alignment of partial amino acid sequences of *Ehrlichia* isolates found in horses, Nicaragua and Brazil. *E. ruminantium* was used as the reference strain in sodB (A) and groEL (B) sequence alignments.
